# N-/T-Type vs. L-Type Calcium Channel Blocker in Treating Chronic Kidney Disease: A Systematic Review and Meta-Analysis

**DOI:** 10.3390/ph16030338

**Published:** 2023-02-22

**Authors:** Mingming Zhao, Ziyan Zhang, Zhiyu Pan, Sijia Ma, Meiying Chang, Jiao Fan, Shunxuan Xue, Yuejun Wang, Hua Qu, Yu Zhang

**Affiliations:** 1 Department of Nephrology, Xiyuan Hospital, China Academy of Chinese Medical Sciences, Beijing 100091, China; 2 Beijing University of Chinese Medicine, Beijing 100029, China; 3 Department of Geriatrics, Zhejiang Aged Care Hospital, Hangzhou Normal University, Hangzhou 310015, China; 4 Xiyuan Hospital, China Academy of Chinese Medical Sciences, Beijing 100091, China; 5 NMPA Key Laboratory for Clinical Research and Evaluation of Traditional Chinese Medicine, Beijing 100091, China; 6 National Clinical Research Center for Chinese Medicine Cardiology, Beijing 100091, China

**Keywords:** N-type calcium channel blocker, T-type calcium channel blocker, L-type calcium channel blocker, chronic kidney disease, proteinuria

## Abstract

Renin-angiotensin system (RAS) inhibitors and calcium channel blockers (CCB) are often used together in chronic kidney disease (CKD). The PubMed, EMBASE, and Cochrane Library databases were searched to identify randomized controlled trials (RCTs) in order to explore better subtypes of CCB for the treatment of CKD. This meta-analysis of 12 RCTs with 967 CKD patients who were treated with RAS inhibitors demonstrated that, when compared with L-type CCB, N-/T-type CCB was superior in reducing urine albumin/protein excretion (SMD, −0.41; 95% CI, −0.64 to −0.18; *p* < 0.001) and aldosterone, without influencing serum creatinine (WMD, −3.64; 95% CI, −11.63 to 4.35; *p* = 0.37), glomerular filtration rate (SMD, 0.06; 95% CI, −0.13 to 0.25; *p* = 0.53), and adverse effects (RR, 0.95; 95% CI, 0.35 to 2.58; *p* = 0.93). In addition, N-/T-type CCB did not decrease the systolic blood pressure (BP) (WMD, 0.17; 95% CI, −1.05 to 1.39; *p* = 0.79) or diastolic BP (WMD, 0.64; 95% CI, −0.55 to 1.83; *p* = 0.29) when compared with L-type CCB. In CKD patients treated with RAS inhibitors, N-/T-type CCB is more effective than L-type CCB in reducing urine albumin/protein excretion without increased serum creatinine, decreased glomerular filtration rate, and increased adverse effects. The additional benefit is independent of BP and may be associated with decreased aldosterone (PROSPERO, CRD42020197560).

## 1. Introduction

Chronic kidney disease (CKD) is defined as a decreased renal function, as shown by a glomerular filtration rate (GFR) of less than 60 mL/(min·1.73 m^2^), or through markers of kidney damage—or both—of at least three months duration, regardless of the underlying cause [1]. CKD has been recognized as a leading public health problem worldwide. The global estimated prevalence of CKD is 13.4% (11.7–15.1%), and patients with end stage renal disease (ESRD) needing renal replacement therapy is estimated between 4.902 and 7.083 million. Through its effect on cardiovascular risk and ESRD, CKD directly affects the global burden of morbidity and mortality worldwide [2]. As the symptoms of CKD occur in the late stages when complications of the disease arise (such as a decline in kidney function and the presence of other comorbidities associated with the disease), the awareness rate of early CKD is low, when in the asymptomatic stage. In the advanced stages of the disease, when kidney function is significantly impaired, patients can only be treated with dialysis or a transplant. With limited treatment options available, an increasing prevalence in both the elderly population, as well as in comorbidities associated with the disease, the prevalence of CKD is set to rise [3]. CKD is an important global health challenge, especially in low- and middle-income countries [4]. National and international efforts for the prevention, detection, and treatment of CKD are needed in order to reduce its morbidity and mortality worldwide.

Proteinuria is an important marker and risk factor for the progression of CKD [5,6]. Hypertension is a common cause of CKD, and since CKD leads to hypertension, hypertension and CKD strongly influence each other. Hypertension is present in approximately 80% of patients with CKD [7]. Antihypertensive therapy may be beneficial in reversing intraglomerular hypertension by reducing hemodynamic damage to the glomeruli and reducing protein filtration [8]. For patients with established CKD, the updated hypertension guidelines have recommended a blood pressure (BP) goal < 130/80 mmHg [9]. A stricter BP target < 130/80 mmHg may help to limit CKD progression, as proteinuria rises [10]. Cardiovascular disease (CVD) is a major cause of morbidity and mortality in patients with CKD. Among patients with CKD and those who possess hypertension without diabetes, intensive BP control is effective in reducing rates of major cardiovascular events and all-cause death [11]. According to the Kidney Disease: Improving Global Outcomes clinical practice guideline, angiotensin-converting enzyme inhibitors (ACEI) or angiotensin-receptor blockers (ARB) are considered first-choice treatment drugs [12]. A large number of evidence has shown that there are voltage-gated calcium channel subtypes in renal vascular and tubular tissues, including L-type, T-type, N-type, and P/Q-type. In addition, the blockade of these calcium channels has resulted in different effects on renal microcirculation [13]. Calcium channel blockers (CCB) targeting voltage-dependent calcium channels are frequently used in combination with renin–angiotensin–aldosterone system (RAAS) inhibitors for CKD patients because of their strong BP-lowering properties and relatively few adverse side effects [14]. When treatment with ACEI/ARB failed to reach the target of BP, ACEI/ARB in combination with CCB should be considered. This treatment has superior benefits on metabolic, renal, and cardiovascular outcomes in patients with hypertension [15,16]. CCB can be divided into L-type (such as amlodipine and nifedipine), L/N-type (such as cilnidipine), L/T-type (such as azelnidipine, efonidipine, mibefradil, etc.), and L/T/N-type (such as benidipine) CCB. In CKD patients with hypertension and proteinuria, the selection of CCB subtypes may be a challenge. The present meta-analysis of randomized controlled trials (RCTs) was designed to assess the effect of N-/T-type CCB on the protection of renal function in CKD patients who were treated with renin-angiotensin system (RAS) inhibitors compared with those treated with L-type CCB. The major aim of this study was to explore the better subtypes of CCB for the treatment of CKD.

## 2. Materials and Methods

### 2.1. Data Sources and Searches

The PubMed, EMBASE, and Cochrane Library databases were searched from inception to August 2022 in order to retrieve relevant articles. Two reviewers (Mingming Zhao and Ziyan Zhang) independently screened the titles and abstracts of all electronic citations, and then searched the full text for comprehensive review and independent re-screening. The disagreements were resolved by consulting a third investigator (Yu Zhang). Medical subject headings and free-text terms used in each database were as follows: “diabetic nephropathy”, “hypertensive nephropathy”, “glomerular disease”, “proteinuria”, “renal insufficiency”, “kidney disease”, “chronic renal failure”, “chronic kidney disease”, “aldosterone”, “N-type calcium channel blocker”, “N-type calcium channel antagonist”, “N-type calcium channel blockade”, “T-type calcium channel blocker”, “T-type calcium channel antagonist”, “T-type calcium channel blockade”, “benidipine”, “cilnidipine”, “azelnidipine”, “efonidipine”, “nilvadipine”, “manidipine”, “mibefradil” (Supplementary Materials File S1).

### 2.2. Study Selection

Inclusion criteria: (1) CKD patients treated with RAS inhibitors; (2) if the intervention group received N-/T-type CCB, and whether the comparison group received L-type CCB; (3) the outcomes involved urine albumin excretion, urine protein excretion, serum creatinine, GFR, BP, and other adverse effects (such as hypotension, hyperglycemia, liver impairment, anemia, skin rash, or hyperkalemia); (4) RCTs; and (5) the articles published in English.

Exclusion criteria: (1) Repeated published studies; and (2) the data were incomplete and the result could not be extracted.

### 2.3. Data Extraction and Quality Assessment

Two reviewers (Mingming Zhao and Ziyan Zhang) independently extracted the data and consulted the third investigator (Yu Zhang) in order to resolve disagreements. The following data were extracted from each of the studies included in our review: the first author’s name, publication year, study design, intervention, sample size, percentage of men, mean age of subjects, duration of intervention, serum creatinine, GFR, urine albumin/protein excretion, systolic blood pressure (SBP), diastolic blood pressure (DBP), and adverse effects. The methodological quality of the included studies was evaluated according to the recommendation of the Cochrane Handbook, including random sequence generation, allocation concealment, blinding of participants and personnel, blinding of outcome assessment, incomplete outcome data, selective reporting, and other biases. A mark of one point was made when the risk was low. A score of 1–3 represents fair quality, and a score of 4–7 represents good quality.

### 2.4. Data Synthesis and Analysis

A random effects model was used to calculate the effect sizes of studies included in our review. For continuous outcomes, we calculated a weighted mean difference (WMD) or standard mean difference (SMD) with a 95% confidence interval (CI). For dichotomous outcomes, we estimated the relative risk (RR) with 95% CI.

Heterogeneity of the included studies was described with the I^2^ index and the Chi-square test. I^2^ < 50% and *p* > 0.05 indicated low heterogeneity. I^2^ ≥ 50% and *p* < 0.05 indicated medium-to-high heterogeneity. A sensitivity analysis was performed to assess the robustness of the pooled results. The potential sources of heterogeneity were detected by meta-regression based on priori selected study characteristics, including urine albumin/protein excretion, the baseline of GFR, the duration of intervention, and the mean age of the subjects. Begg’s test and Egger’s test were used to evaluate the publication bias. Statistical analysis was performed using Stata (version 15.1). The methodological quality was assessed by RevMan5.3. Additionally, we have registered the protocol for the present systematic review and meta-analysis; the registration number in PROSPERO is CRD42020197560.

## 3. Results

### 3.1. Characteristics and Quality of the Studies

**Figure 1:**
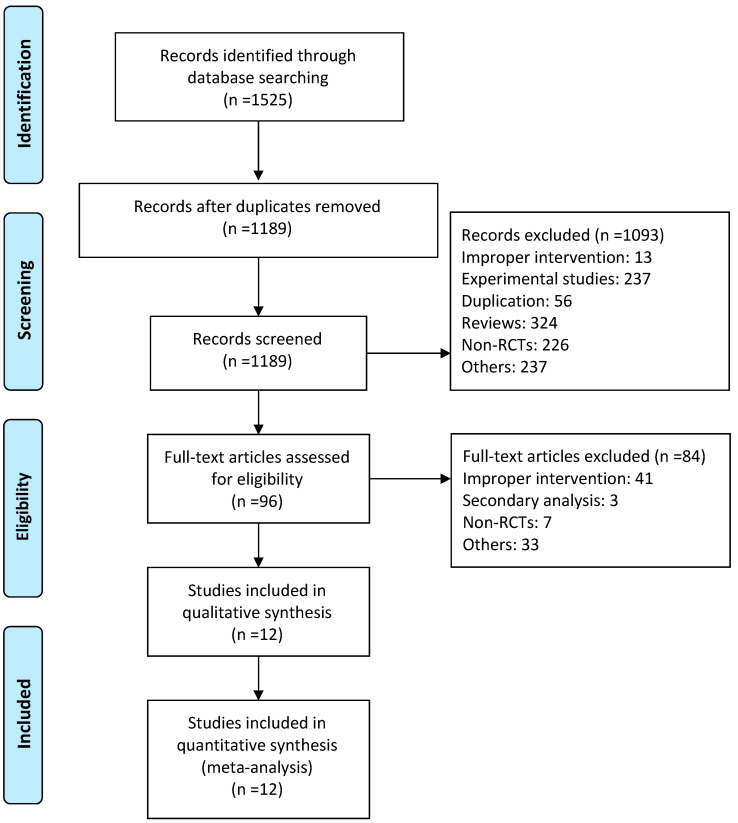
Flow diagram of searching for eligible studies.

**Figure 2:**
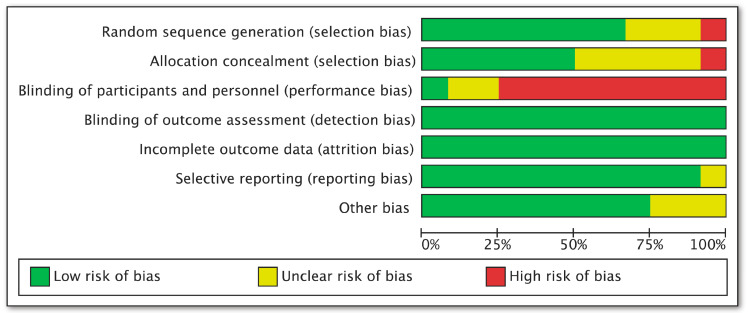
Risk of bias summary.

A total of 1525 studies (361 from PubMed, 1051 from EMBASE, and 113 from the Cochrane Library) were identified. After reading the abstract and full text, 12 studies were determined in the end to meet the inclusion criteria (Figure 1). The characteristics of the individual trials are presented in Table 1. Twelve parallel arm RCTs included 967 CKD patients treated with RAS inhibitors. The sample size varied from 30 to 339. The mean age of the subjects ranged from 31.60 to 72.90 years, and the duration of intervention ranged from six to 12 months. Three studies enrolled patients with a GFR ≥ 60 mL/min or mL/(min·1.73 m^2^) and seven studies enrolled patients with GFR < 60 mL/min or mL/(min·1.73 m^2^). Two studies did not report the subjects’ baseline renal function. One study was of fair quality (score 1–3) and 11 were of good quality (score 4–7) (Figure 2) [17].

### 3.2. Effect of N-/T-Type CCB vs. L-Type CCB on Urine Albumin/Protein Excretion

**Figure 3:**
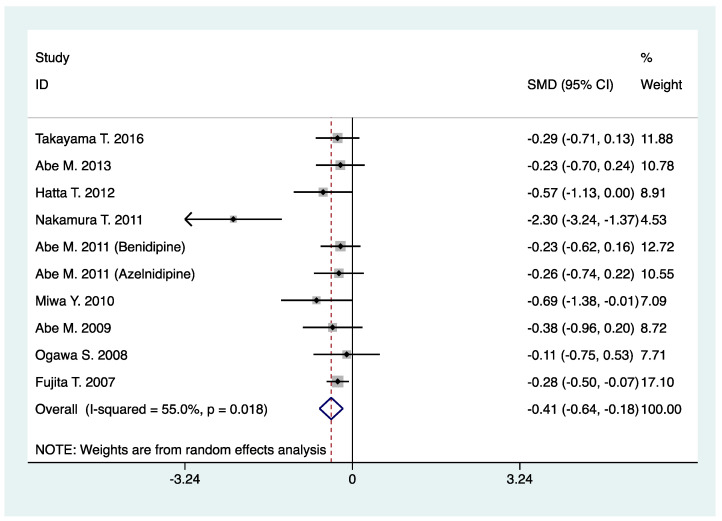
Comparison of N-/T-type CCB vs. L-type CCB in terms of urine albumin/protein excretion in CKD patients treated with RAS inhibitors (mg/g of creatinine or mg/24 h).

Urine albumin excretion as an efficacy end point was reported in five trials (367 patients), with all studies reporting a urine albumin/creatinine ratio (mg/g creatinine). Urine protein excretion as an efficacy end point was reported in five trials (466 patients), with two studies reporting a urine protein/creatinine ratio (mg/g creatinine), two studies reporting 24-h urine protein (mg/24 h), and one study reporting a urine protein/creatinine ratio (mg/g creatinine) or 24-h urine protein (mg/24 h). When compared with L-type CCB, N-/T-type CCB significantly reduced urine albumin/protein excretion in CKD patients who were treated with RAS inhibitors (SMD, −0.41; 95% CI, −0.64 to −0.18; *p* < 0.001) (Table 2, Figure 3).

### 3.3. Effect of N-/T-Type CCB vs. L-Type CCB on Serum Creatinine and GFR

**Figure 4:**
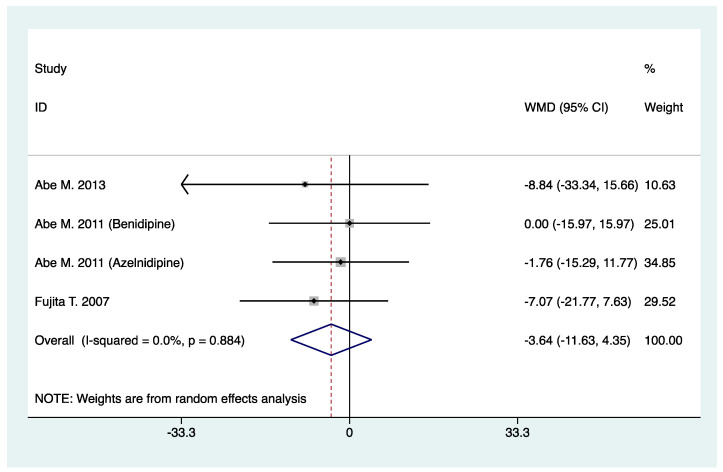
Comparison of N-/T-type CCB vs. L-type CCB for serum creatinine in CKD patients treated with RAS inhibitors (μmol/L).

**Figure 5:**
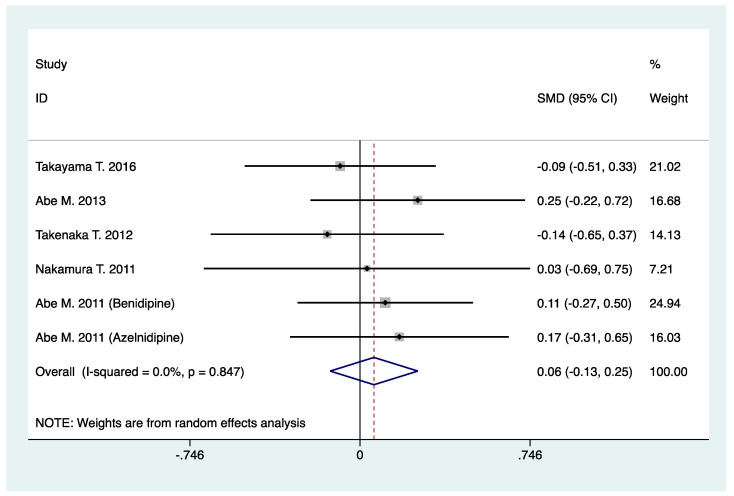
Comparison of N-/T-type CCB vs. L-type CCB for GFR in CKD patients treated with RAS inhibitors (mL/min or mL/min/1.73 m^2^).

Four studies involving 580 patients reported the results of serum creatinine, and six studies involving 418 patients reported the results of GFR (GFR was calculated by using equations as defined in individual trials). By meta-analysis, N-/T-type CCB in combination with RAS inhibitors was not associated with increased serum creatinine (WMD, −3.64; 95% CI, −11.63 to 4.35; *p* = 0.37) (Table 2, Figure 4), neither was decreased GFR observed (SMD, 0.06; 95% CI, −0.13 to 0.25; *p* = 0.53) when compared with L-type CCB in combination with RAS inhibitors (Table 2, Figure 5).

### 3.4. Effect of N-/T-Type CCB vs. L-Type CCB on BP

**Figure 6:**
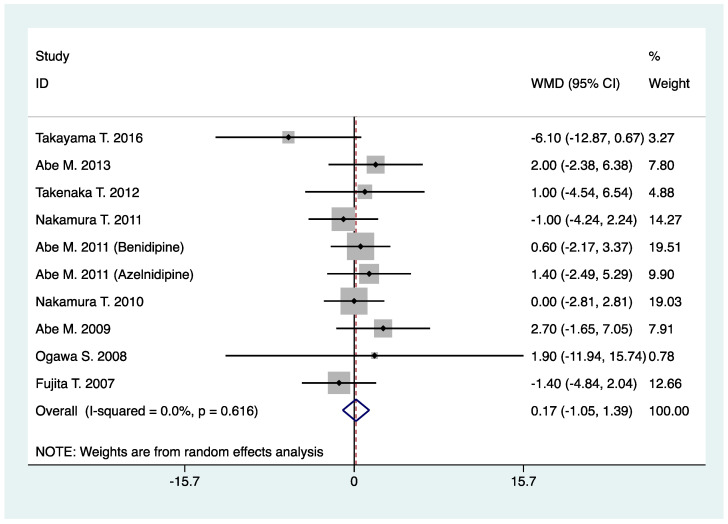
Comparison of N-/T-type CCB vs. L-type CCB for SBP in CKD patients treated with RAS inhibitors (mmHg).

**Figure 7:**
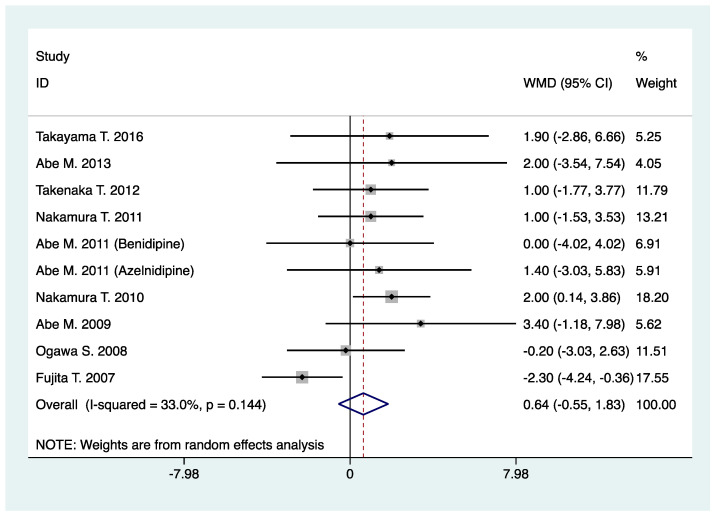
Comparison of N-/T-type CCB vs. L-type CCB for DBP in CKD patients treated with RAS inhibitors (mmHg).

As shown in Table 2, N-/T-type CCB relative to L-type CCB yielded no significant effect on SBP (WMD, 0.17; 95% CI, −1.05 to 1.39; *p* = 0.79) (Table 2, Figure 6) and DBP (WMD, 0.64; 95% CI, −0.55 to 1.83; *p* = 0.29) in CKD patients treated with RAS inhibitor (Table 2, Figure 7).

### 3.5. Effect of N-/T-Type CCB vs. L-Type CCB on Plasma Aldosterone

There were three studies that reported plasma aldosterone levels in CKD patients treated with RAS inhibitors. One study demonstrated that the plasma aldosterone percent changes from the baseline were significantly decreased in the cilnidipine group when compared with the amlodipine group at the 48-week time point [19]. Two studies demonstrated that there were no significant changes in plasma aldosterone levels in the amlodipine group. However, the plasma aldosterone levels were decreased in the benidipine and azelnidipine groups [23,24].

### 3.6. Safety Analysis

**Figure 8:**
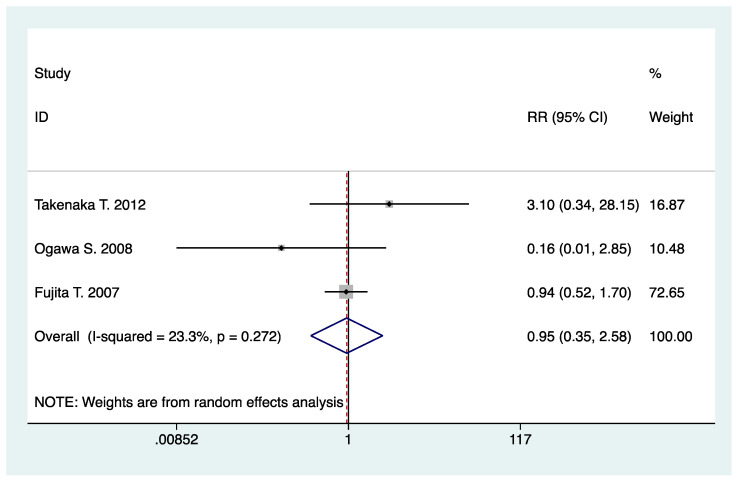
Comparison of N-/T-type CCB vs. L-type CCB in terms of adverse effects in CKD patients treated with RAS inhibitors.

Adverse events, such as hypotension, palpitation, dizziness, ankle edema, etc., occurred in three studies. There were no significant effects regarding N-/T-type CCB in combination with RAS inhibitors with respect to adverse effects (RR, 0.95; 95% CI, 0.35 to 2.58; *p* = 0.93) (Table 2, Figure 8). Seven of the included studies reported no adverse events, and two studies did not report adverse events.

### 3.7. Sensitivity Analysis and Meta-Regression

Significant heterogeneity was observed for urine albumin/protein excretion (Table 2). In order to ensure reliability of the present meta-analysis, we evaluated the robustness of the result of urine albumin/protein excretion using sensitivity analysis, which indicated that the result of urine albumin/protein excretion was robust.

We detected the potential sources of heterogeneity using a meta-regression analysis, based on priori selected study characteristics, including urine albumin/protein excretion, the baseline of GFR, the duration of intervention, and the mean age of subjects. A significant heterogeneity was observed for the outcome of urine albumin/protein excretion, which was dependent on the mean age of the subjects (exp, 1.05; 95% CI, 1.01 to 1.10; *p* = 0.03). By using a meta-regression analysis, it was found that the heterogeneity of urine albumin/protein excretion was not associated with urine albumin/protein excretion, the baseline of GFR, and the duration of intervention.

### 3.8. Publication Bias

Begg’s test and Egger’s test were used to evaluate publication bias, based on the key outcomes of the trials included in the meta-analysis. The results indicated less susceptibility to publication bias, except for urine albumin/protein excretion (Table 2).

## 4. Discussion

In the present meta-analysis of the 12 RCTs encompassing 967 CKD patients treated with RAS inhibitors, we aimed to assess the effect of N-/T-type CCB on the protection of renal function when compared with L-type CCB. The quality of the included studies in this meta-analysis was relatively good. The results demonstrated that N-/T-type CCB was superior to L-type CCB in reducing urine albumin/protein excretion and aldosterone, without increased serum creatinine, decreased GFR, and increased adverse effects. Nonetheless, N-/T-type CCB did not decrease the BP when compared with L-type CCB.

CKD is both a common cause of hypertension and a complication of uncontrolled hypertension. The interaction between hypertension and CKD is complex and increases the risk of adverse cardiovascular and cerebrovascular outcomes. This is particularly significant in the setting of resistant hypertension commonly seen in patients with CKD [30]. Albuminuria or proteinuria can be used as a marker of progressive CKD and can also be used as an indicator for the initiation of hypertension treatment by regulating the RAAS with ACEI or ARB [31,32]. There is a variety of mechanisms involved in the development and progression of renal fibrosis, but glomerular injury leading to proteinuria may form a critical loopback effect between proteinuria and tubulointerstitial injury. If proteinuria persists, it can lead to tubular cell damage, resulting in tubulointerstitial inflammation and fibrosis, thereby leading to a decline in renal function and to the progression of CKD toward ESRD [8].

Sustained hypertension can lead to worsening kidney function. In addition, progressive decline in kidney function can conversely lead to worsening BP control [33]. More strict BP control is associated with lower mortality in patients with hypertension and CKD [34,35]. It is usually necessary to use a combination of antihypertensive medications to achieve target BP [36,37]. CCB has a certain application value in the treatment of hypertension in CKD, which can improve the BP control in patients with proteinuria who have been treated with RAS inhibitors, and may also play an additional renal protective role [38]. There are voltage-gated calcium channel subtypes in renal vascular, including L-type, T-type, N-type, and P/Q-type. In CKD patients with hypertension and proteinuria, the selection of CCB subtypes may be a challenge. The systemic hypotensive effect of CCB can decrease the glomerular pressure. CCB inhibits L-type calcium channels, which exist in glomerular afferents but not efferent arterioles, as well as in glomerular hypertension caused by afferent arteriole-specific vasodilation. N-type CCB suppresses the release of norepinephrine from the sympathetic nerve terminal by blocking of N-type calcium channels, as well as in dilate afferent and efferent arterioles that are innervated sympathetically, thus resulting in decreased glomerular pressure and acting as a renoprotective agent [39,40]. T-type calcium channels are widely distributed in both afferent and efferent arterioles. The inhibition of T-type calcium channels dilates afferent and efferent arterioles, as well as alleviates glomerular damage [41]. It has been reported that L-type, N-type, and T-type calcium channels are also localized in the glomerulus and tubules. There are two types of calcium channels, L-type and T-type, which have been reported to localize in the mesangial cells. Further, glomerular podocytes express N-type calcium channels [42,43]. L-type and T-type calcium channels exist in the luminal side of the distal tubules [44]. T-type calcium channels are also located in the inner medullary collecting ducts, distal collecting ducts, and collecting tubules [45]. N-type and T-type CCB can improve glomerular and tubular injury independently of hypotensive effect, which is superior to L-type CCB [14,46]. Cinidipine is an L/N-type CCB, which can reduce BP, inhibit the increase in blood urea nitrogen and the decrease in creatinine clearance, and improve glomerulosclerosis in Dahl salt-sensitive rats [47]. Cilnidipine can also suppress the development of proteinuria greater than amlodipine possibly through inhibiting N-type calcium channel-dependent podocyte injury in spontaneously hypertensive rat/ND mcr-cp. Compared with amlodipine, cilnidipine can prevent the increase in desmin staining in glomerular podocytes, as well as restore the expression of podocin and nephrin [48]. Efonidipine is an L/T-type CCB, which can reduce SBP and proteinuria in spontaneously hypertensive rats and has a renal protective effect, which depends not only on hemodynamic effects, but also on the inhibition of Rho-kinase activity, tubulointerstitial fibrosis, and epithelial–mesenchymal transitions [49]. In the deoxycorticosterone acetate-salt model regarding the high glomerular blood pressure salt model, compared with amlodipine, L/T-type CCB mibefradil provides better nephroprotection including amelioration of proteinuria and glomerular injury. Furthermore, this protection does not depend on systemic and glomerular antihypertensive responses [50]. Despite treatment with ACEI and ARB, many studies have shown that the renin-angiotensin cascade is not completely blocked. The therapeutic blockade of renin-angiotensin but persistent or elevated plasma aldosterone levels is called an “aldosterone escape” [51,52]. Aldosterone has the effect of increasing fibrosis, collagen deposition, inflammation, as well as the remodeling of the heart and blood vessels [53]. “Aldosterone escape” can cause the formation of renal sodium retention and edema in various diseases, including cardiovascular diseases, cirrhosis, and kidney diseases, as well as lead to disease progression [54,55,56,57]. Aldosterone is recently considered to be an aggravating factor of renal diseases, and its secretion from the adrenal gland is mediated by T-type calcium channels [58]. In addition, T-type CCB ameliorates renal dysfunction by inhibiting inflammatory process and renin secretion [59,60]. Taken together, the kidney is endowed with a variety of calcium channel subtypes, which have different effects on renal microcirculation. In addition to the reliable antihypertensive effect, the inhibition of N-type and T-type calcium channels may provide additional benefits in the progression of CKD through nonhemodynamic mechanisms [61].

According to our results, although serum creatinine and GFR were not improved, N-/T-type CCB was more effective in reducing urine albumin/protein excretion than L-type CCB in the RAS inhibitor-treated patients with CKD. No significant differences in SBP and DBP were noted between N-/T-type CCB and L-type CCB. Therefore, the additional benefit of N-/T-type CCB in reducing urine albumin/protein excretion was independent of BP. The inhibitory effect of N-/T-type CCB on aldosterone may play a role in reducing urine albumin/protein excretion. Except for efficacy, adverse effects are another important factor to be considered. In this meta-analysis, the safety of N-/T-type CCB was equivalent to that of L-type CCB. Therefore, N-/T-type CCB is more suitable as a second-line antihypertensive drug for CKD patients treated with RAS inhibitors.

CCB is a common antihypertensive drug in the treatment of kidney diseases. More and more researchers have begun to explore the application of different types of CCB. However, there are only a few meta-analyses that have been performed in order to analyze the efficacy and safety of the use of N-/T-type CCB compared with L-type CCB in CKD patients treated with RAS inhibitors. This meta-analysis evaluated the effects of N-/T-type CCB on urine albumin/protein excretion, serum creatinine, GFR, BP, plasma aldosterone, and other adverse events. However, there are certain limitations to this study. First, only 12 RCTs met the inclusion criteria, most of which include a relatively small population. The design of the included studies needs to be improved. For example, the two included studies did not report adverse events. In order to further clarify the application prospect of N-/T-type CCB in combination with RAS inhibitors in CKD, further large-scale and high-quality research is needed. Second, due to few eligible RCTs, subgroup analysis was not performed based on subtypes of CCB, including N-type CCB and T-type CCB. With the increase in CCB research in the future, the efficacy of N-type CCB and T-type CCB can be further compared. Third, the included studies were heterogeneous; we conducted sensitivity analysis and meta-regression analysis in order to ensure the reliability of this meta-analysis. Fourth, the population of included studies were mainly patients with CKD stage 1–3. Whether the conclusions of this study are applicable to patients with CKD stage 4–5 needs further clinical trials in order to prove. The follow-up period also needs to be extended in order to evaluate the long-term follow-up results, such as ESRD or death.

## 5. Conclusions

In conclusion, in CKD patients treated with RAS inhibitors, N-/T-type CCB is more effective than L-type CCB in reducing urine albumin/protein excretion without increased serum creatinine, decreased GFR, and increased adverse effects. The additional benefit is independent of BP and may be associated with decreased aldosterone.

## Figures and Tables

**Table 1:** Characteristics of eligible studies included in this meta-analysis.

Studies	CCB		N (T/C)	Male (%)		Age (Y)		Duration (Months)	sCr (umol/L)	GFR [mL/min or mL/(min·1.73 m^2^)]	Urine Albumin or Protein Excretion (mg/g Creatinine or mg/24 h)	SBP (mm Hg)	DBP (mm Hg)
	N-/T-Type CCB	L-Type CCB		T	C	T	C						
Takayama T. 2016 [18]	Benidipine + RASI	Amlodipine + RASI	41/47	73.20	66.00	68.30	72.90	12	NR	54.25	250.00 ^†^	135.75	70.70
Abe M. 2013 [19]	Cilnidipine + ARB	Amlodipine + ARB	35/35	54.29	60.00	66.00	67.00	12	101.66	47.00	323.50 ^†^	143.00	81.50
Takenaka T. 2012 [20]	Azelnidipine + ARB	Amlodipine + ARB	29/30	63.00	60.00	66.00	67.00	12	NR	25.50	NR	139.50	84.50
Hatta T. 2012 [21]	Cilnidipine + ARB	L-type CCB + ARB	24/26	54.17	88.46	57.00	56.00	12	164.42	36.05	1595.00	128.90	71.80
Nakamura T. 2011 [22]	Azelnidipine + ARB	Amlodipine + ARB	15/15	60.00	60.00	45.30	45.50	6	NR	81.90	1000.00	155.00	92.50
Abe M. 2011 (Benidipine) [23]	Benidipine + ARB	Amlodipine + ARB	52/52	57.69	57.69	67.30	67.50	6	109.62	44.80	≥30 ^†^	144.50	81.50
Abe M. 2011 (Azelnidipine) [24]	Azelnidipine + Olmesartan	Amlodipine + Olmesartan	34/33	61.76	60.61	65.80	66.00	6	93.70	53.80	383.50 ^†^	140.70	81.65
Nakamura T. 2010 [25]	Benidipine + ACEI/ARB	Amlodipine + ACEI/ARB	20/20	55.00	55.00	33.50	31.60	12	63.21	92.35	1550.00	153.50	91.50
Miwa Y. 2010 [26]	Cilnidipine + ACEI/ARB	Amlodipine + ACEI/ARB	18/17	50.00	52.94	66.80	57.90	12	76.47	NR	490.00	138.25	75.90
Abe M. 2009 [27]	Benidipine + ARB	Amlodipine + ARB	24/23	62.50	65.22	65.90	65.50	6	266.97	21.95	3364.50	153.55	86.95
Ogawa S. 2008 [28]	Azelnidipine + ACEI/ARB	Nifedipine + ACEI/ARB	21/17	52.38	52.94	61.70	59.40	12	68.07	69.80	490.69 ^†^	156.50	78.45
Fujita T. 2007 [29]	Cilnidipine + ACEI/ARB	Amlodipine + ACEI/ARB	179/160	67.60	58.13	59.90	59.30	12	113.15	NR	1816.50	152.40	87.45

Abbreviations: CCB, calcium channel blocker; N, number of patients; T, treatment group; C, control group; Y, year; sCr, serum creatinine; GFR, glomerular filtration rate; SBP, systolic blood pressure; DBP, diastolic blood pressure; RASI, renin-angiotensin system inhibitor; ACEI, angiotensin-converting enzyme inhibitor; ARB, angiotensin-receptor blocker; and NR, not reported. ^†^ Value represents urinary albumin excretion.

**Table 2:** Summary effect of N-/T-type CCB vs. L-type CCB in CKD patients treated with RAS inhibitors.

Outcome	No. Studies	No. Participants	Random Effects Model		Assessment of Heterogeneity		Publication Bias (*p*-Value)	
			95% CI	*p*-Value	I^2^ (%)	*p*-Value	Begg’s Test	Egger’s Test
Urine albumin/protein excretion (mg/g of creatinine or mg/24 h)	10	833	SMD: −0.41 (−0.64, −0.18)	<0.001	55.0	0.02	0.03	0.10
Serum creatinine (μmol/L)	4	580	WMD: −3.64 (−11.63, 4.35)	0.37	0.0	0.88	1.00	0.52
Glomerular filtration rate (mL/min or mL/(min·1.73 m^2^))	6	418	SMD: 0.06 (−0.13, 0.25)	0.53	0.0	0.85	0.71	0.87
Systolic blood pressure (mmHg)	10	882	WMD: 0.17 (−1.05, 1.39)	0.79	0.0	0.62	0.86	0.91
Diastolic blood pressure (mmHg)	10	882	WMD: 0.64 (−0.55, 1.83)	0.29	33.0	0.14	0.47	0.31
Adverse effects	3	442	RR: 0.95 (0.35, 2.58)	0.93	23.3	0.27	1.00	0.92

## Data Availability

All data generated or analyzed during this study are included in this published article and its supplementary information files.

## Supplementary Materials

The following supporting information can be downloaded at: https://www.mdpi.com/article/10.3390/ph16030338/s1, File S1. PubMed, EMBASE, and Cochrane Library search strategies.
