# Supplementary material for: N-/T-Type vs. L-Type Calcium Channel Blocker in Treating Chronic Kidney Disease: A Systematic Review and Meta-Analysis
Source: Pharmaceuticals (Basel). 2023 Feb 22;16(3):338. doi: 10.3390/ph16030338 (PMC10053533; doi:10.3390/ph16030338)
Supplement: Supplementary file 1 [file pharmaceuticals-16-00338-s001.zip › pharmaceuticals-2120447-supplementary/Supplementary Materials File S1.pdf]

PubMed, EMBASE, and Cochrane Library search strategies.

Databases	Search	Search Strings	Hits (2022.8.16)
PubMed	#1	((((((((diabetic nephropathy [MeSH Terms]) OR hypertensive nephropathy [MeSH Terms]) OR glomerular disease [MeSH Terms]) OR proteinuria [MeSH Terms]) OR renal insufficiency [MeSH Terms]) OR kidney disease [MeSH Terms]) OR chronic renal failure [MeSH Terms]) OR chronic kidney disease [MeSH Terms])	576,032
	#2	aldosterone [All Fields]	50,997
	#3	#1 or #2	619,053
	#4	((((((((((((N-type calcium channel blocker) OR (N-type calcium channel antagonist)) OR (N-type calcium channel blockade)) OR (T-type calcium channel blocker)) OR (T-type calcium channel antagonist)) OR (T-type calcium channel blockade)) OR (benidipine)) OR (cilnidipine)) OR (azelnidipine)) OR (efonidipine)) OR (nilvadipine)) OR (manidipine)) OR (mibefradil) [All Fields]	5,829
	#5	#3 and #4	361
Cochrane Library	#1	MeSH descriptor: [Diabetic Nephropathies] explode all trees	1,561
	#2	(hypertensive nephropathy): ti,ab,kw	383
	#3	(glomerular disease): ti,ab,kw	6,167
	#4	MeSH descriptor: [Proteinuria] explode all trees	2,410
	#5	MeSH descriptor: [Renal Insufficiency] explode all trees	9,840
	#6	MeSH descriptor: [Kidney Diseases] explode all trees	17,459
	#7	MeSH descriptor: [Kidney Failure, Chronic] explode all trees	4,888
	#8	MeSH descriptor: [Renal Insufficiency, Chronic] explode all trees	7,347
	#9	#1 or #2 or #3 or #4 or #5 or #6 or #7 or #8	22,892
	#10	(aldosterone): ti,ab,kw	5,297
	#11	#9 or #10	27,494
	#12	(N-type calcium channel blocker): ti,ab,kw	54
	#13	(N-type calcium channel antagonist): ti,ab,kw	18
	#14	(N-type calcium channel blockade): ti,ab,kw	10
	#15	(T-type calcium channel blocker): ti,ab,kw	50
	#16	(T-type calcium channel antagonist): ti,ab,kw	20
	#17	(T-type calcium channel blockade): ti,ab,kw	7
	#18	(benidipine): ti,ab,kw	88
	#19	(cilnidipine): ti,ab,kw	165
	#20	(azelnidipine): ti,ab,kw	136
	#21	(efonidipine): ti,ab,kw	42
	#22	(nilvadipine): ti,ab,kw	85
	#23	(manidipine): ti,ab,kw	75
	#24	(mibefradil): ti,ab,kw	87

	#25	#12 or #13 or #14 or #15 or #16 or #17 or #18 or #19 or #20 or #21 or #22 or #23 or #24	680
	#26	#11 and #25	113
EMBASE	#1	'diabetic nephropathy'/exp OR 'hypertensive nephropathy'/exp OR 'glomerulopathy'/exp OR 'proteinuria'/exp OR 'kidney failure'/exp OR 'kidney disease'/exp OR 'chronic kidney failure'/exp	1,208,457
	#2	aldosterone	102,554
	#3	#1 or #2	1,289,098
	#4	'n type' AND calcium AND channel AND blocker OR ('n type' AND calcium AND channel AND antagonist) OR ('n type' AND calcium AND channel AND blockade) OR ('t type' AND calcium AND channel AND blocker) OR ('t type' AND calcium AND channel AND antagonist) OR ('t type' AND calcium AND channel AND blockade) OR benidipine OR cilnidipine OR azelnidipine OR efonidipine OR nilvadipine OR manidipine OR mibefradil	8,020
	#5	#3 and #4	1,051
